# CRISPR/Cas9 Targeted Mutagenesis for Functional Genetics in Maize

**DOI:** 10.3390/plants10040723

**Published:** 2021-04-08

**Authors:** Charles T. Hunter

**Affiliations:** Chemistry Research Unit, USDA Agricultural Research Service, Gainesville, FL 32608, USA; Charles.Hunter@usda.gov

**Keywords:** gene editing, gene knockout, polycistronic gRNA, transformation vector

## Abstract

The CRISPR/Cas9-based system for targeted mutagenesis has become an indispensable tool for functional genetics in plants. CRISPR/Cas9 allows users to generate loss-of-function alleles in genes of interest with precision and in a simple-to-use system. This manuscript outlines important points to consider for experimental design and utilization of CRISPR/Cas9 in targeted mutagenesis in maize. It also introduces the pRGEB32-BAR vector modified for use in maize that allows simultaneous delivery of multiple gRNAs using a simple assembly. Vector selection, gRNA design, genetic strategies, and genotyping approaches are discussed, with an emphasis on achieving isolation of homozygous mutant plants in a time- and cost-efficient manner.

## 1. Overview

The emergence of precision DNA nuclease tools, and CRISPR/Cas9 in particular, has made gene editing for targeted gene disruption accessible for even small laboratories. The simplicity of vector design and assembly means that researchers with little technical experience in molecular cloning or genetic engineering can generate targeted mutations in genes of interest for use in functional genetic characterization. The increased access and feasibility of using CRISPR for functional genetics does not mean that the process is necessarily rapid, simple, or inexpensive, however. One major bottleneck for using CRISPR-based gene disruption in maize has been access to transformation protocols or services, but rapid expansion in these areas is occurring. Other points of constraint are centered around the cost, time, and space required to isolate useful CRISPR-generated alleles in the needed combinations and appropriate genetic backgrounds for functional analyses. These less-considered, but important, constraints are discussed here, and recommendations are offered for streamlining the development of experiment-ready plants for functional genetic studies.

First demonstrated as a functional tool in mammalian cells in 2012 [1], CRISPR/Cas9 has since taken the world of molecular genetics by storm. Utilized in many different species of plants and animals, CRISPR has become a universal tool for targeted genetic manipulation, whether it be gene disruption, gene replacement, or more complex applications. Many excellent reviews cover the mechanics of CRISPR biochemistry [2,3], the varied applications of CRISPR [4,5], and technical considerations for using CRISPR, including gRNA design and vector construction/delivery [6,7]. While the potential uses of CRISPR are incredibly varied and exciting, by far the most frequent use of CRISPR/Cas9 in plants is as a gene editing tool for causing loss-of-function knockout alleles. Here the focus will be on practical considerations for using CRISPR in such a gene disrupting context as a resource for functional genetics in plants, particularly in maize.

Some basic concepts regarding CRISPR biology and use need to be introduced to facilitate this discussion. The CRISPR/Cas9 toolset is a two-component system composed of a DNA nuclease (Cas9) that uses a separate single-stranded guide RNA (gRNA) to recognize and cut specific DNA sequences. By stably or transiently expressing Cas9 and gRNAs designed to bind target DNA sequences, it is possible to cause double-stranded cuts precisely at desired locations. As DNA repair machinery in a cell “fixes” the DNA break through non-homologous end joining, small deletions or insertions that lead to gene disruption result. To utilize CRISPR for functional genetics, gRNAs are designed to recognize the target gene(s) and expressed alongside Cas9. If successful, mutations in the target gene(s) result, providing loss-of-function alleles for genetic characterization.

Importantly, the use of CRISPR for functional genetics in a system like maize is a time- and resource-intensive undertaking, largely due to the demands of growing generations of plants and the genotyping needed to isolate desired alleles in relevant combinations for experimentation. Identification and isolation of effective alleles in inbred backgrounds and in the desired combinations can require multiple generations and a daunting amount of genotyping to track alleles. Multiple loss-of-function alleles for each gene target are needed so that observed phenotypes can be confidently associated with gene disruption rather than linked loci. Before properly-controlled experiments can take place, the Cas9-expressing transgene should be eliminated by genetic selection and mutant alleles should be isolated as homozygotes in inbred backgrounds. Conducting plant transformation in inbred lines (like B104 in maize) is highly desirable so that wildtype control plants are available for phenotypic comparisons to mutants.

## 2. Considerations for Experimental Design

**Figure 1:**
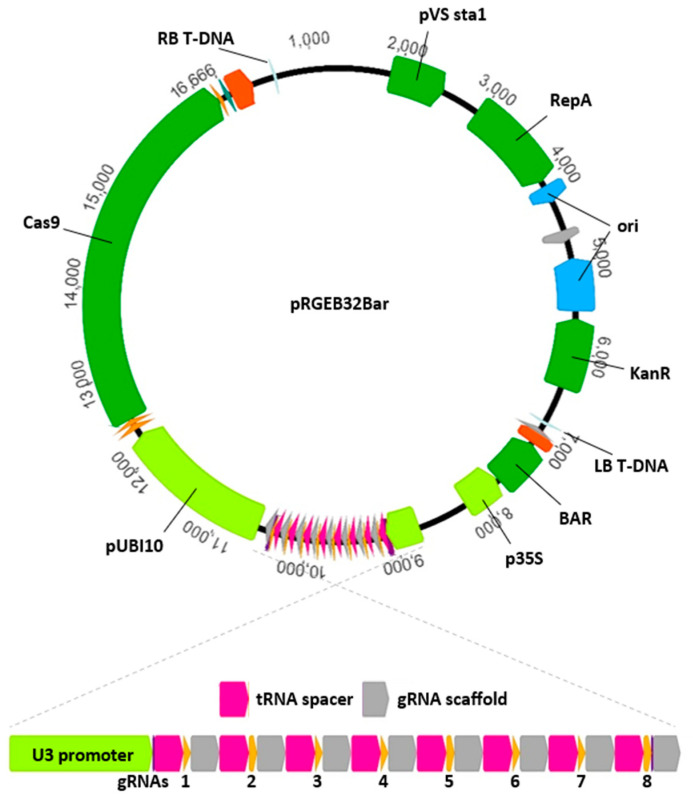
Diagram of the pRGEB32Bar CRISPR/Cas9 delivery vector for use in maize. The vector was modified from pRGEB32 by replacing the hygromycin selectable marker with the BAR gene that confers Basta resistance and is preferred for maize transformation. The remaining components of the vector were retained from the original pRGEB32 vector and function in both rice and maize. Components between the left (LB) and right (RB) borders constitute the transfer DNA that will be stably incorporated into the host genome. This includes expression cassettes for BAR, Cas9, and the polycistronic gRNA assembly. The rice Pol-III-driven promoter U3 drives expression of a polycistronic tRNA CRISPR assembly composed of repeated units of tRNA spacers, 20-nt CRISPR gRNAs, and gRNA scaffolds. A single transcript from this assembly will be processed by plant tRNA processing machinery to produce mature gRNA molecules. In the example presented here, the transcript will be processed into 8 mature gRNAs, each targeting a unique genomic location.

Choosing a competent delivery vector is important when using CRISPR/Cas9 for functional genetics. A variety of strategies and vectors have been successfully used in maize [8,9,10], and each may have benefits. A custom vector modified from pRGEB32 (gifted by Yinong Yang) for use in maize is described here. The pRGEB32 vector [11] was designed to deliver Cas9, a selectable marker, and multiple gRNAs in agrobacterium-mediated transformation of rice. The vector allows for multiplexed gRNA expression by leveraging the plant’s tRNA processing machinery to produce multiple functional gRNAs from a single polycistronic tRNA and CRISPR gRNA transcript. The vector expresses three components important for functionality in maize: (1) Cas9 from *Streptococcus pyogenes* driven by the rice ubiquitin promoter; (2) a custom polycistronic gRNA assembly (precursor for one or more gRNAs) driven by the rice U3 snoRNA (Pol III) promoter; and (3) HPTII (hygromycin resistance) driven by the 35S promoter. Advantages of expressing multiple gRNAs in a single transcript include simplicity of vector design and construction and the use of only a single gRNA-expressing promoter. The pRGEB32 vector used here was modified by replacing the hygromycin selectable marker with the BAR gene (confers glufosinate-ammonium resistance), facilitating this vector’s use in maize. The modified vector is available on Addgene.com under pRGEB32-BAR (Figure 1). Addition of gRNA cassettes is relatively simple with the pRGEB32-based vector. The BsaI cloning sites allow oligonucleotide/Gateway-based assembled or synthetic dsDNA flanked by properly oriented BsaI recognition sites to be added via restriction digest and annealing [11]. Synthetic genes (from Genscript.com, capable of synthesizing highly repetitive dsDNA) containing up to 10 independent gRNAs with tRNA linker sequences have been cloned into the modified pRGEB32-BAR vector in a single digestion/ligation. The vector has been utilized for the delivery of over 30 gRNAs targeting various maize genes in 10 independent CRISPR/Cas9 delivery vectors, demonstrating the utility of the vector in maize, including the functionality of the rice ubiquitin and U3 promoters and the rice tRNA sequence. A similar multiplexed gRNA system using maize promoters and tRNAs in a pCAMBIA3301 vector has also been demonstrated to function in maize [12].

The delivery of multiple gRNAs simultaneously accomplishes two important goals: (1) targeting potentially redundant genes; and (2) saving on transformation costs. By targeting all members of a gene family using a single transgene, double, triple, or higher-order mutants can be obtained more quickly than by stacking multiple independently-derived mutants through crossing and selection. In many crop species, and particularly in maize with its relatively recent genome duplication [13,14], gene redundancy is typical, and disruption of multiple paralogous genes is commonly needed to achieve loss of activity for a targeted enzymatic reaction. Additionally, plant transformation currently represents the bulk of the costs associated with CRISPR-based gene disruption (over $4500 per vector in inbred B104 maize), so targeting multiple, even unrelated, genes simultaneously and then segregating alleles away from one another in future generations can help reduce costs on a per-gene basis. This comes with a trade-off as increasing the number of targets leads to more genotyping requirements and usually additional generations to isolate desired alleles. Targeting three or four genes simultaneously represents a good middle ground for optimizing cost and keeping genotyping demands reasonable.

## 3. Designing gRNAs

One of the advantages of using CRISPR/Cas9 lies in the flexibility regarding potential target sites. Generally, any 20-nucleotide sequence followed by an NGG protospacer adjacent motif (PAM) is a viable target, so there are effectively never cases where CRISPR cannot target genomic regions. Important considerations for gRNA design include location within the target gene, presence of targeted regions in gene splice variants, multi-gene targeting for single gRNAs, and potential off-target binding sites. To improve the chances of obtaining complete loss of function alleles, gRNAs are usually targeted near the 5-prime region of genes and in coding sequence retained in all splice variants. Redundant genes can often be targeted in conserved regions by a single gRNA, though 100% sequence identity along 23 nucleotides that terminate in an NGG is a major constraint. There are a variety of web-based, free-to-use tools available to assist in design and selection of gRNAs in plants, including CHOPCHOP (chopchop.cbu.uib.no [15,16]) and CRISPR-P (cirspr.hzau.edu.cn/CRISPR2/ [17,18]), both of which can query the maize genome for target specificity to help avoid off-target sites. These tools provide users with ranked gRNA candidates based on algorithms that consider GC-content, predicted secondary structure, and gRNA target specificity. By using these web-based tools and considering placement within genes and multi-gene targeting, the best-suited gRNAs can be selected. It is beneficial to select two gRNAs separated by around 100 bases for each target gene. This helps assure that effective alleles will be obtained even if one of the two gRNAs do not function and it also improves the possibility of causing large deletions of the sequence between gRNAs [10,11,12]. While rare, larger deletions are desirable because they are simple to track via PCR and gel electrophoresis alone compared to the very small deletions that require more complex genotyping strategies (see below).

**Figure 2:**
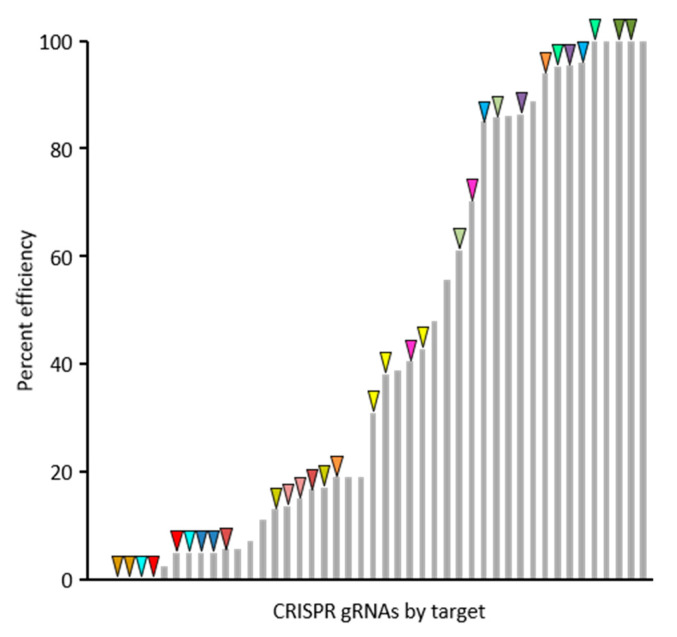
Efficiency rates of 30 gRNAs in maize. The proportion of target alleles edited in T_0_ plants varies widely by gRNA and target, though the distribution of efficiency rates is skewed towards the extremes, with 39% of gRNAs tested having less than 15% efficiency and 30% having greater than 85% efficiency. Guide RNAs that target multiple genes (indicated by same-color triangles) tend to show similar efficiency rates for each target, indicating that gRNA efficiency is largely determined by characteristics of the gRNA itself and less so by characteristics of the target sequence.

For the purposes of this communication, a summary of 30 gRNAs success rates and efficiencies are presented, though the target genes are not discussed as functional analyses for these genes are ongoing. The web-based tool CHOPCHOP (v3) was used to select best-suited gRNAs for targeted mutagenesis of 20 candidate genes and Agrobacterium-based plant transformation was conducted in B104 embryonic callus at Iowa State University Plant Transformation Facility. Screening of first generation transgenic (T_0_) plants by PCR and Sanger sequencing revealed the occurrence of edits for the majority of gRNA binding sites. Only 4 out of 30 gRNAs did not result in detected edits in T_0_ plants. The efficiency rates for the 30 gRNAs is shown in Figure 2 and was calculated based on the proportion of edited gene copies out of those surveyed. The efficiency rates varied from 0% to 100% and was not correlated with the position of the gRNA in polycistronic pre-gRNA transcripts. Dual-targeted gRNAs, those that targeted more than one gene, tended to be effective at both targets (same-color arrows in Figure 2), showing that gRNA efficiency is largely determined by the characteristics of the gRNA itself. However, occasional instances of varying efficiencies between two targets for gRNAs with multiple targets shows that characteristics of the genomic gRNA binding site can also influence efficiency.

## 4. Choosing Priority Alleles

When selecting edits identified in T_0_ plants that will be isolated and employed in functional genetic studies, it is critical to consider the impact of edits on gene translation. Insertions and deletions in multiples of three typically result in amino acid changes but not in nonsense-coding frameshift mutations. Indels in multiples of three are likely to retain activity and are undesirable for the purposes of creating loss-of-function alleles. Though 1-nucleotide deletions represent the most common mutations, it is important to screen many alleles and select the best-suited for functional analyses. The occurrence of 3x-base indels that retain activity can also be a benefit of using CRISPR. When a gene’s function is critical to survival of a T_0_ plant or for gamete development, partially- or fully-functional alleles allow for plant survival and recovery of seeds containing the transgene, even with 100% gRNA efficiency. Bi-allelic plants with one 3x-base indel and one knock-out allele are common. In such cases, the effects of losing gene activity can still be examined by self-pollination of heterozygous plants and examination of seeds, embryos, or offspring segregating for the non-functional allele.

Another consideration for choosing which edits to pursue becomes relevant when using two gRNAs for a single gene. Most often, both gRNAs will result in small edits and not a large deletion between gRNA binding sites, which can complicate genotyping and introduces the chance of frameshift-reversing mutations. These can occur where a frameshift mutation at the site of gRNA1 is returned to in-frame by a mutation at gRNA2. Such alleles are not ideal selections for candidate loss-of-function alleles as a portion of the translated protein remains normal and may retain partial or full activity. Even if effective-looking mutations occur at the gRNA1 target site; it is important to assay the gRNA2 target site for mutations to confirm that the two linked edits do not sum to a multiple of three.

## 5. Isolation of CRISPR/Cas9-Derived Alleles for Functional Genetic Studies

**Figure 3:**
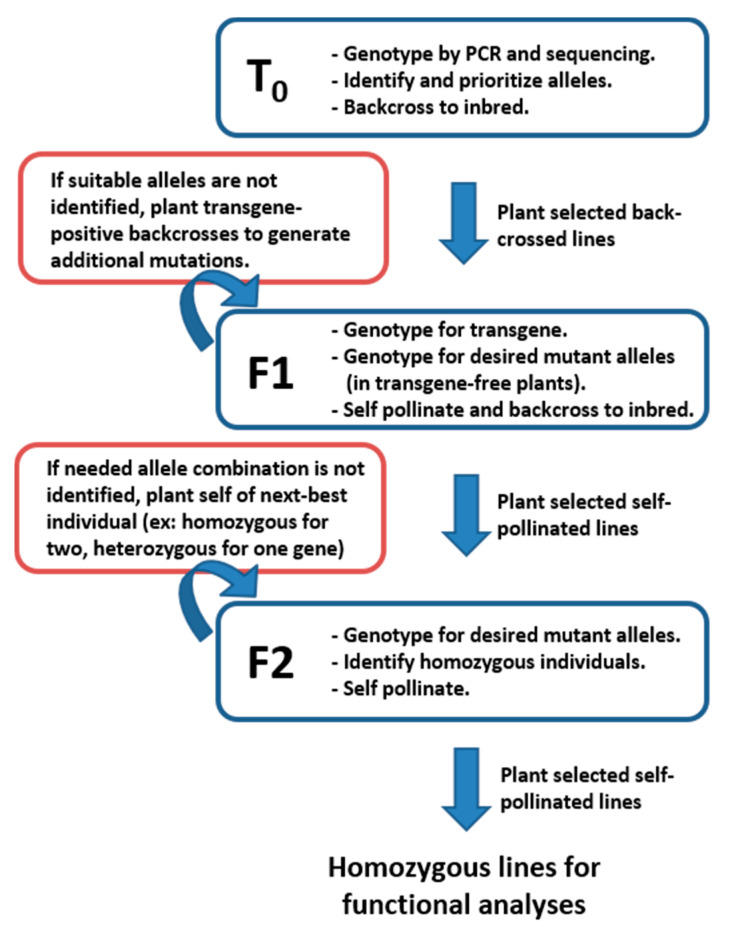
Typical work-flow for field genetics and genotyping strategy to achieve homozygous mutant lines for functional characterization. Three generations are usually needed to achieve transgene-free, homozygous mutants in the desired combination for experimentation. Initially, T_0_ plants are screened for mutations-of-interest and backcrossed to the appropriate inbred line. The resulting F1 plants are grown, self-pollinated, and backcrossed again. The F1 plants are first genotyped for the absence of the transgene and plants negative for the transgene are genotyped for the target allele(s). Seeds resulting from self-pollination of transgene-free, heterozygous plants are grown and genotyped to identify homozygous mutants. These are self-pollinated to produce stable, transgene-free, homozygous lines to be used in functional genetic testing.

To isolate CRISPR-derived alleles as homozygous and transgene-free, three generations of plants are usually required after Cas9-driven allele generation in T_0_ plants. Figure 3 shows the typical workflow to achieve desired combinations for use in functional genetic testing. Initial allele identification is conducted by PCR amplification and Sanger sequencing of regions surrounding expected edits in T_0_ plants. Mutations identified in T_0_ plants are typically germ-line stable and heritable in future generations. Genotyping all T_0_ plants (typically around 50 individuals) usually results in numerous alleles in each of the target genes. At least two effective alleles, usually the largest deletions in non-multiples of three, are selected for future isolation as homozygotes and experimentation. The T_0_ plants carrying the selected alleles are back-crossed to the wildtype inbred and the resulting F1 seed provides the basis for allele isolation in the next two generations. In cases where suitable alleles for functional characterization are not identified in T_0_ plants, additional screening for mutant alleles in F1 plants can be conducted. Occasionally gRNAs have low efficiency rates and it is necessary to genotype many plants to find suitable alleles.

F1 offspring of plants containing selected alleles for characterization are genotyped to identify plants containing the desired alleles and not containing the Cas9-expressing transgene. The F1 plants are expected to segregate 1:1 for transgene absence or presence because the initial transgene is usually present as a single-copy heterozygote in T_0_ plants. Genotyping F1 plants by PCR for the presence of the transgene, followed by genotyping for the chosen alleles in the target genes, allows for identification of transgene-free plants containing the desired alleles. It is critical to remove the transgene before progressing to mutant isolation as expression of Cas9 and gRNAs may continue to cause new mutations in F1 or later generations, which can complicate genotyping and confuse future experiments. Self-pollination of transgene-free plants produces F2 segregating families wherein homozygous, non-transgenic mutants in the desired combinations can be identified. The most important consideration in determining the number of F1 plants needed is the number of genes targeted by the transgenic construct. For a single allele, and assuming normal gamete transmission, 25% of plants are expected to have the desired combination of transgene-free and target allele positive. If more alleles were targeted simultaneously and need to be isolated separately as single mutants or stacked (double mutants, triple mutants, etc.), the number of needed F1 plants increases exponentially. For example, if alleles in three different target genes are all present in a T_0_ plant, F1 plants from a backcross of that plant are expected to contain any specific combination of alleles at a rate of 1:16, so more F1 plants need to be grown and genotyped.

Segregating F2 families containing selected alleles and absent the transgene are genotyped for the desired allele combinations, usually homozygous mutations in the target genes. Again, the number of plants needed to obtain the desired combination of alleles scales with the number of target genes present in a given line. If more than a few gene mutants are being stacked to create triple, quadruple, or higher-order mutants, it is unlikely that the homozygous mutants will be identified in the initial F2 family. In these cases, planting self-pollinated ears from individuals containing all desired alleles and fixed as homozygous in as many possible will allow isolation of correct combinations in later generations.

## 6. Genotyping Strategies

**Figure 4:**
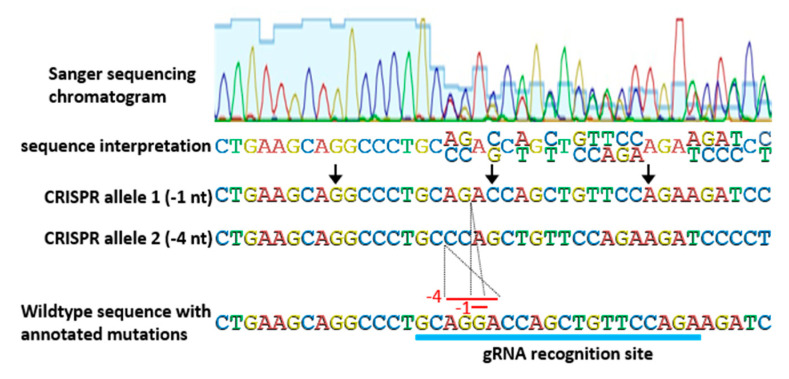
Determining sequences of bi-allelic PCR products directly from Sanger sequencing chromatograms. The chromatogram shows a zoom-in of the relevant portion of a longer Sanger sequencing product. Sanger sequencing chromatograms display the four DNA nucleotides as peaks of different colors and when multiple peaks are shown at the same position (overlapping), it indicates a bi-allelic PCR product. Sequence interpretation from such a convoluted chromatogram is still possible, as each position with overlapping peaks can be assigned two bases at that position, corresponding to the two DNA sequences in the PCR product. Because CRISPR most typically results in small deletions, examining the sequence interpretation for occurrence of the wildtype sequence allows each position to be defined for one allele. Direct interpretation of CRISPR-derived mutations in biallelic PCR products avoids the time and expense of subcloning.

Genotyping strategies for identifying and then tracking CRISPR-derived alleles are another critical consideration. Allele identification of CRISPR-based edits is not always as straight forward as simple readouts of Sanger sequencing of PCR products. Very often, T_0_ plants are bi-allelic (containing different mutations in one or both gene copies) and PCR amplification results in two similar, but not exact, products that cannot be separated on an agarose gel. In most cases, bi-allelic PCR products can still be interpreted by careful examination of Sanger sequencing chromatograms, despite two distinct DNA fragments being present (Figure 4). Mutations can be pinpointed to the position in a Sanger sequencing chromatogram at which convoluted, overlapping, double peaks appear. At that position, the two PCR products diverge, but because CRISPR-based mutations are typically small deletions or insertions, the normal gene sequence is retained, only shifted. By aligning sequencing data with genomic sequence, mutations are usually discernable. In some cases, where more complex mutations have occurred, or the sequencing data is unclear, subcloning of PCR products is needed to obtain unambiguous results.

While Sanger sequencing of PCR products can be costly (between $2 and $5 per reaction) and time-consuming, it is the most reliable method for tracking alleles and provides certainty that the intended allele is being tracked. Some alleles, particularly large (>30 bases) deletions or insertions can be distinguished on agarose gels, which can save time and expense when alleles need to be tracked across many individuals and multiple generations. It may be worthwhile to screen many transgene-positive F1 plants for uncommon, large, easy-to-genotype alleles before advancing to isolation of alleles in desired combinations for functional studies. For mutations that cannot be distinguished on agarose gels, higher-throughput and less expensive methods compared to Sanger sequencing of PCR products are available, including high-resolution melting curve analysis [19], fluorescent PCR capillary electrophoresis [20], hetero/homo-duplex agarose gel electrophoresis [21], and polyacrylamide gel electrophoresis [22]. These methods are in theory sensitive enough to distinguish PCR products with even very small changes, including 1-nucleotide deletions/insertions. However, they require time-consuming assay development and validation and they cannot provide the precise nature of detected edits.

In cases where very large numbers of transgenic plants or gRNA targets need to be genotyped, high-throughput sequencing approaches can be most efficient for initial allele identification in T_0_ plants or tracking alleles in segregating populations [23,24,25]. There are now several genomics and high-throughput sequencing companies, including GENEWIZ and CD Genomics among others, that offer services to genotype and validate CRISPR/Cas9-induced mutations. As sequencing and library preparation costs continue to lower, these high-throughput sequencing approaches are likely to become the most direct and cost-effective methods for genotyping CRISPR-induced alleles. For the time being however, when relatively few samples are being screened and some uncertainty exists regarding alleles that may be present, direct sequencing of PCR products provides the most information with the highest certainty.

## 7. Concluding Remarks

By using the CRISPR/Cas9 system, researchers can target any gene or gene family for mutagenesis, representing an incredible leap forward for functional genetics. The technology is powerful, simple to use, and accessible, providing a precise and flexible tool for researchers that far surpasses mutagenesis resources of the past. Still, it is important to have realistic expectations regarding costs, timelines, and likely outputs for CRISPR-based mutagenesis. Considering the points discussed herein prior to initiating CRISPR-based projects will increase efficiency and help prevent unnecessary mistakes.
